# Effects of mental contrasting on sleep and associations with stress: A randomized controlled trial

**DOI:** 10.1177/13591053231159168

**Published:** 2023-03-15

**Authors:** Laura I Schmidt, Andreas B Neubauer, Martin Stoffel, Beate Ditzen, Jana Schirmaier, Claudia Farrenkopf, Monika Sieverding

**Affiliations:** 1 Ruprecht-Karls-University Heidelberg, Germany; 2 DIPF | Leibniz Institute for Research and Information in Education, Germany

**Keywords:** cortisol awakening response, mental contrasting with implementation intentions, sleep duration, sleep quality, stress

## Abstract

Mental contrasting with implementation intentions (MCII) has been successfully applied to improve health-related behaviors (e.g. exercise). We explored its effectiveness to improve sleep outcomes beyond effects of sleep hygiene (SH) information, and investigated associations with stress. Eighty university employees (mean age: 29.6, SD = 4.5) were randomized to either a MCII + SH or a SH-only condition. During a baseline-week and a post-intervention week, sleep duration (Fitbit Alta and self-report), sleep quality, and stress were assessed daily and saliva was collected to assess the cortisol awakening response (CAR). In total, self-reported sleep quality and duration increased, but there was no meaningful condition*week interaction for sleep parameters or CAR. Higher average stress was associated with shorter sleep duration and lower sleep quality. Within-person, days with higher stress were followed by nights with lower sleep quality. Despite overall improvements, effects of MCII were not confirmed. MCII might be less effective to improve behaviors which are less controllable.

## Introduction

Insufficient sleep is a widespread problem and the consequences are well documented and far-reaching. For adults between 18 and 64 years, the National Sleep Foundation (NSF) generally recommends a range of nightly sleep duration from 7 to 9 hours (Hirshkowitz et al., 2015). Epidemiological evidence links insufficient sleep to increased likelihood of depression, obesity, physical inactivity, cardiovascular disease or diabetes mellitus, and meta-analytic evidence demonstrates increased all-cause mortality due to shorter sleep duration (e.g. Cappuccio et al., 2011). Regarding performance, meta-analyses found impairments due to sleep disruption for simple but also complex cognitive tasks (i.e. Wickens et al., 2015). Hence, it is not surprising that insufficient sleep has been repeatedly associated with worse performance in the academic and working environment (e.g. Litwiller et al., 2017).

Psychological interventions to improve sleep in academia very rarely assess objective sleep outcomes and largely focus on early undergraduate or college students (Friedrich and Schlarb, 2018). However, high prevalence rates for insufficient sleep have also been reported by (post-)graduate students and the general population. In representative German working samples, 31% reported poor sleep at least three times a week (Marschall et al., 2017), more than a third failed to reach the 7-hour benchmark, and 66% reported to sleep less than they needed on workdays (NSF, 2013). Hence, with a focus on non-clinical samples without manifest insomnia, the development of non-pharmacological interventions for young working populations is important to reduce risks that have been found for burnout, depressive symptoms, or days of incapacity to work (Marschall et al., 2017).

Existing intervention studies aiming to improve sleep in healthy adults most commonly provide information on the importance of sleep or on behavior promoting good sleep, so-called sleep hygiene practices (Friedrich and Schlarb, 2018). There is evidence, that brief and easy-to-implement interventions based on sleep hygiene information can have positive effects on sleep-related outcomes in non-clinical samples (Albakri et al., 2021; Kakinuma et al., 2010). As effect-sizes remained rather small, that is Cohen’s *d* = 32 for sleep hygiene interventions on sleep duration in a meta-analysis (Friedrich and Schlarb, 2018), we aimed to test a self-regulatory intervention based on mental contrasting with implementation intentions (MCII) against the effects of sleep hygiene information only.

### Implementation intentions and sleep

The formulation of implementation intentions is a post-intentional behavior change technique fostering the translation of intention into behavior which has been applied in interventions based on the Theory of Planned Behavior (Ajzen, 1991). Implementation intentions are action plans that specify when, where, and how a particular behavior will be performed and include an explicit conditional statement in the form of “If (cue x) occurs, then I will perform (behavior y)” (Gollwitzer and Sheeran, 2006). Behaviors become more automatic, as the need for conscious decision-making is reduced by the mental stimulus-response linkage between cue and behavior. There is robust evidence regarding effects of implementation intentions across various health behaviors (Gollwitzer and Sheeran, 2006), but so far only three studies have applied implementation intentions to sleep. Loft and Cameron (2013) used a modified imagery version of implementation intentions among employees (*N* = 104) and found larger improvements in sleep hygiene behavior and self-reported sleep quality, in comparison to arousal reduction and control imagery. Mairs and Mullan (2015) compared the effect of implementation intentions on sleep hygiene and self-reported sleep quality with the behavior change technique of self-monitoring among students (*N* = 72). In both study groups, sleep quality was higher at post-intervention measurement 2 weeks after baseline. Only regarding one sleep hygiene behavior, namely “avoiding stress and anxiety-provoking activities,” implementation intentions outperformed self-monitoring. Both studies did not assess (objective) sleep duration. In a third study (Schmidt et al., 2023) on teachers (*N* = 69), participants in the intervention group were guided to develop implementation intentions. During a 3-week intervention period, all participants (including the active control group) wore activity trackers to measure sleep duration and kept diaries to assess sleep quality. After 7 weeks, results indicated medium-sized to large effects for sleep quality and sleep duration favoring the intervention group.

### Mental contrasting

Mental contrasting is a pre-intentional self-regulation technique that uses cognitive and motivational mechanisms to facilitate strong goal commitments or goal intentions (Oettingen and Gollwitzer, 2010), which are essential for the success of implementation intentions. First, individuals name an important and feasible wish. Second, they vividly imagine the best outcome. Third, they identify a central inner obstacle to realizing that wish and again, imagine that obstacle. The combined strategy of mental contrasting and implementation intentions (MCII) has recently been applied across health behaviors (e.g. physical activity, nutrition), and a meta-analysis of seven MCII interventions indicated a small-to-medium effect (Hedges’ *g* = 0.28) on different health domains (Cross and Sheffield, 2019). To our knowledge, MCII had not been transferred to sleep when our study was conducted. A study published after our data collection examined the effects of MCII on bedtime procrastination in two RCTs (Valshtein et al., 2020). A reduced discrepancy between intended and actual bedtime was reported, but objective measures for sleep were not assessed.

### Stress and poor sleep

A secondary objective of our study was to investigate associations between daily stress and sleep outcomes, as well as possible additional intervention effects on subjective stress and the cortisol awakening response (CAR). Self-reported stress has been linked to both subjective and objective sleep quality. For example, Hall et al. (2015) found in their large-scale longitudinal study that participants in a high stress group reported more sleep complaints compared to moderate/low stress groups. Additionally, in-home polysomnography revealed lower sleep efficiency. Similarly, but in a micro-longitudinal approach across 42 days, Åkerstedt et al. (2012) reported that increased stress at bedtime was associated with poorer sleep quality. Moreover, there is evidence linking poor sleep to altered diurnal cortisol slopes and an altered CAR (Stalder et al., 2016). Although evidence regarding the direction of these effects is mixed, most studies reported that poor sleep was associated with an attenuated diurnal cortisol secretion, that is, flattened slopes and reduced CAR’s (Strahler et al., 2017). This is in line with an emerging picture that links increased chronic stress to *decreased* diurnal cortisol secretion (Law and Clow, 2020). So far, no sleep-based intervention has focused on assessing changes in diurnal measures of the HPA axis, such as the CAR.

### Study aims and hypotheses

The present study aimed to expand the self-regulation technique of MCII to the context of sleep outcomes, namely sleep duration and sleep quality, in a non-clinical population. Our main objective was to compare the effects of sleep hygiene information only (SH) with the combined effects of MCII + SH regarding objective and subjective sleep parameters. We hypothesized that:

(1) MCII + SH increase fitbit-measured sleep duration more than SH only,
(2) MCII + SH increase self-reported (subjective) sleep duration more than SH only,
(3) MCII + SH increase self-reported (subjective) sleep quality more than SH only.

Moreover, we aimed to analyze associations of daily stress with sleep outcomes:

(4) Self-reported daily stress is negatively associated with sleep outcomes (subjective sleep quality, fitbit-measured, and self-reported sleep duration). On the within-person level, this effect reflects shorter sleep duration and lower sleep quality on nights after days with higher than usual stress levels.

As exploratory hypotheses, we expected stronger increases in the CAR in the MCII + SH group, as compared to the SH-only group, as well as a stronger decrease in self-reported stress in the MCII + SH group compared to the SH-only group.^1^

## Method

Data were analyzed using R version 4.2.1 for Windows. Multilevel models were estimated using the nlme package (Pinheiro et al., 2021). Statistical significance was determined at α = 0.05 (two-tailed). The study was pre-registered (https://osf.io/q2te4) and followed APA ethical standards and the 1964 Helsinki declaration and its amendments.

### Recruitment and sample

Early career researchers, that is, doctoral candidates and scientific staff, employed at Heidelberg University were invited via e-mail and newsletters to participate if they had the intention to improve their sleep. To approach the statistical power for the multilevel models, we approximated the effect size that can be detected with a power of 80% using a sensitivity analysis in G*Power (Faul et al., 2009) for a 2 × 2 mixed ANOVA. For a sample of *N* = 80, adequate power would be obtained to detect a true population effect of *f* = 0.16, corresponding to an η² of 0.025. A total of 80 individuals met the inclusion criteria (sufficient German language, no psychiatric disorder or sleep medication) and were included. Mean age was 29.6 years (SD = 4.5), 62% were women, 9% had children, and 50% lived together with their partner. Participants did not receive monetary compensation, but were offered feedback on their CAR and the book “Why We Sleep” by Matthew Walker.

### Procedure

**Figure 1.**
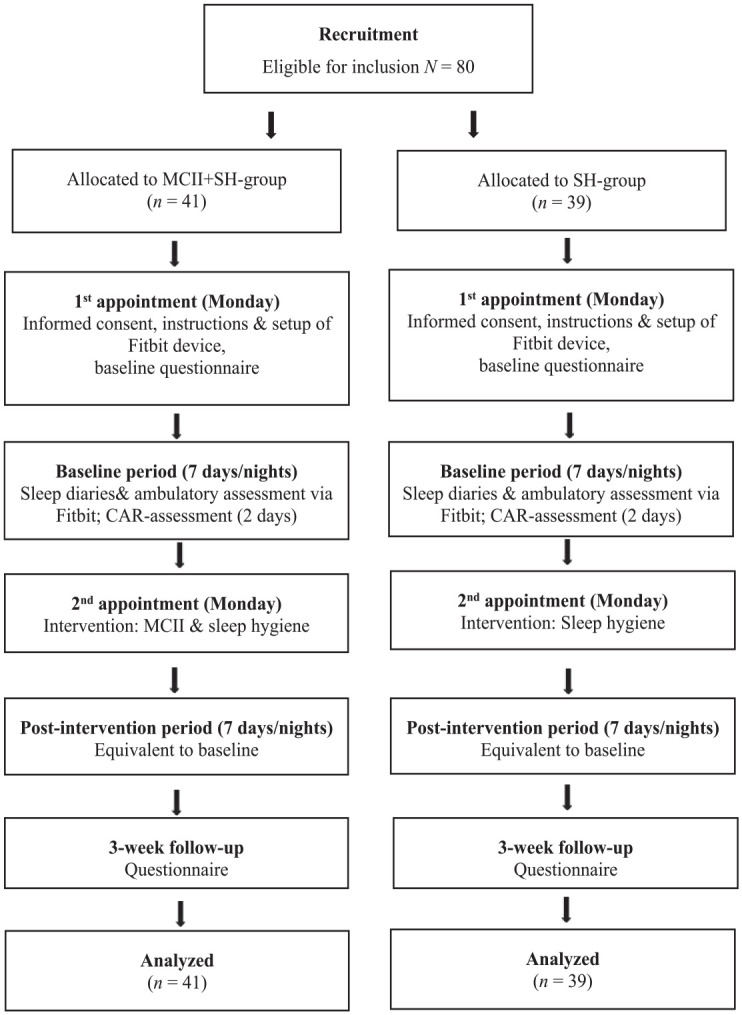
Procedure and flow of participants.

The present study compared two intervention conditions (MCII + SH vs SH) and analyzed associations between stress and sleep outcomes and in a single-blinded, randomized controlled trial with daily/nightly assessments in a baseline-week and analog daily/nightly assessments in a post-intervention week (Figure 1). The authors JS or CF generated the random allocation sequence by tossing a coin. On the first day of the baseline-week (first appointment, Monday), participants received information on the study procedure, informed consent was obtained, a Fitbit device was handed out and connected to the smartphone, and a link to the baseline questionnaire (SoSci Survey) was provided. This questionnaire assessed sociodemographic and health information (e.g. somatic or psychiatric disorders), the German version of the Pittsburgh Sleep Quality Index (PSQI; Backhaus et al., 2002), bedtime procrastination (Kroese et al., 2016), control variables for the CAR, and the average stress level for the past 2 weeks (HEI-STRESS; Schmidt et al., 2018) which was also assessed at a follow-up 3 weeks post-intervention. On each day of the baseline-week, participants answered morning and evening questionnaires and wore their Fitbit each night. On the next Monday (second appointment), the intervention (MCII + SH or SH) was conducted in face-to-face settings in groups of two to six participants, followed by the post-intervention week with identical assessments. Saliva was collected on days four and five of the baseline and the intervention week (Thursdays and Fridays). On each day, participants provided three samples, starting immediately upon awakening (S1), 30 minutes (S2), and 45 minutes (S3) thereafter.

### Intervention

Participants in the SH-group (*N* = 39) were instructed to read a handout on sleep hygiene practices provided by the German Sleep Society. Afterwards, questions were answered, the information was discussed, and a list of sleep hygiene behaviors was distributed, as well as links to websites with further information on sleep hygiene. The delivery of the SH information lasted 15–20 minutes. Participants in the MCII + SH condition (*N* = 41) received the same information plus the MCII intervention directly afterwards (+15–20 minutes). They were asked to specify and write down their most relevant wish with regard to sleep (e.g. go to bed earlier). Participants were encouraged to formulate realistic wishes, such that their expectations of success would be sufficiently high for MCII to be effective. Afterwards, they were instructed to imagine the most positive consequences of fulfilling their wish, followed by identification of the most relevant obstacle.

After this, participants were asked to formulate three implementation intentions, by specifying how, where and when they could act to (a) prevent their obstacle from occurring, (b) overcome their obstacle in case it occurred, and (c) specify another goal-directed behavior to improve their sleep. Examples were given for each step. Finally, participants received a handout and were asked to repeat the MCII procedure on their own on the following day.

### Measurements of the main study variables

*Sleep duration* was measured using the Fitbit Alta HR (certification 2014/53/EU), a commercially available tracker based on 3-axis accelerometry and heart rate tracking and additionally assessed via self-report. Participants were first asked to indicate their subjective sleep duration every morning in the morning questionnaire. Afterward, they copied their fitbit-measured sleep duration to the questionnaire. The two measures for sleep duration were highly correlated (within-person level: *r* = 0.73; between-person: *r* = 0.76), indicating good construct validity.

*Sleep quality* was assessed as the mean of two items: the first item derived from the Pittsburgh Sleep Diary (Monk et al., 1994) asking participants in the daily morning questionnaire to rate their sleep quality on a visual analog scale from 0 to 100. In the second item, participants rated the extent to which they felt alert after awakening (0–100). The two items were correlated at the within-person level, *r* = 0.41, and the between-person level, *r* = 0.63, and averaged into one indicator of subjective sleep quality per person and day.

*Stress* was assessed with two items in the daily evening questionnaire. Participants rated how stressed they felt that day and how often they had the opportunity to relax. Both items were answered on visual analog scales from 0 (not at all) to 100 (very stressed/very often) and were averaged with the second item reversed (within-person level: *r* = 0.53; between-person: *r* = 0.16). Higher values indicate higher subjective stress levels.

### Assessment of the CAR

Salicaps^®^ (IBL, Hamburg, Germany) sampling tubes were used to collect saliva for the analysis of salivary cortisol (sCort) using the “passive drool” method. Several precautions to increase data quality were taken (see Stalder et al., 2016). Participants were instructed to refrain from eating, drinking, smoking, brushing their teeth, and physical activity during the post-awakening period (45 minutes) and to store the samples in their refrigerator as soon as possible. Immediately after providing each sample (S1, S2, and S3), participants were asked to report on state-level variables (e.g. caffeine intake) and control items (e.g. medication during the night) potentially influencing the CAR. The area under the curve with respect to increase (AUCi) was calculated and interpreted (Pruessner et al., 2003).

### Laboratory analysis and preprocessing of CAR data

Saliva samples were stored at −80°C. The concentration of sCort (ng/mL) was analyzed using an enzyme-linked immunosorbent assay at the Institute of Medical Psychology in Heidelberg, Germany. The inter-assay coefficient of variation (CV) was 5.46% and the intra-assay CV was 2.91%. Following Stalder et al. (2016), potentially problematic data were discarded. Specifically, we removed data from participants who were pregnant (*N* = 1), worked in nightshifts (*N* = 3), or had a medical condition that might affect cortisol levels (*N* = 9). For women, we removed data on samples collected between 13 and 17 days after the onset of their last menstruation (*n* = 32). Further, data were removed if S1 was taken >15 minutes after awakening (*n* = 18), if sleep medication was taken (*n* = 2), or if any food, caffeine, or alcohol had been consumed (*n* = 7). This resulted in a final sample of 185 CAR estimates obtained from 63 participants.

### Data analysis

Data were analyzed using multilevel modeling to account for the nested data structure with repeated measurements (Level 1) nested within individuals (Level 2). For our hypotheses targeting intervention effects, individual *j*’s observation on day *i* (*Y_ii_*; depending on the model, this was either fitbit-measured sleep duration, subjective sleep duration, or sleep quality) was predicted by group (SH = 0 vs MCII + SH = 1), week (baseline = 0 vs post-intervention = 1), and the group*week interaction. Additionally, we controlled for gender (*sex_j_*; male = 0; female = 1) and day of the week (*dow_ij_*; night during the week = 0; weekend night = 1). A random slope of the effect of week was estimated to account for potential within-group heterogeneity in the change from before to after the intervention.

Formal model description:

Level 1: (within participants)

(1) $$Y_{ij}=\beta_{0j}+\beta_{1j}\cdot {\mathrm{week}}_{ij}+\beta_{2j}\cdot {\mathrm{dow}}_{ij}+\varepsilon_{ij}$$

Level 2: (between participants)

(2) $$\beta_{0j}=\gamma_{00}+\gamma_{01}\cdot {\mathrm{group}}_{j}+\gamma_{02}\cdot {\mathrm{sex}}_{j}+\upsilon_{0j}$$

(3) $$\beta_{1j}=\gamma_{10}+\gamma_{11}\cdot {\mathrm{group}}_{j}+\upsilon_{1j}$$

(4) $$\beta_{2j}=\gamma_{20}$$

For the hypotheses on the association between daily stress and sleep parameters, the dependent variable (fitbit-measured sleep duration, subjective sleep duration, or sleep quality) was predicted by stress reported on the previous evening (*stress_(i_*_−1)*j*_) as well as an individual’s average stress reported across the whole observation period (*stress_j_).* Time-varying stress reports were centered on the person-mean, and the average stress of an individual was centered on the grand mean of all daily stress reports. With this centering, the effect of the time-varying predictor can be interpreted as the pure within-person effect, and the effect of the average stress reports represents the pure between-person effect of stress on the dependent variable (Wang and Maxwell, 2015).

Level 1: (within participants)

(5) $$Y_{ij}=\beta_{0j}+\beta_{1j}\cdot {\mathrm{stress}}_{\left(i-1\right)j}+\beta_{2j}\cdot {\mathrm{dow}}_{ij}+\varepsilon_{ij}$$

Level 2: (between participants)

(6) $$\beta_{0j}=\gamma_{00}+\gamma_{01}\cdot {\mathrm{stress}}_{j}+\gamma_{02}\cdot {\mathrm{sex}}_{i}+\upsilon_{0j}$$

(7) $$\beta_{1j}=\gamma_{10}+\upsilon_{1i}$$

(8) $$\beta_{2j}=\gamma_{20}$$

## Results

Three observations were set to missing, since time stamps indicated that morning assessments were filled in prior to the evening assessments of the previous day.^2^ Table 1 depicts descriptive statistics separately for the two groups. The two sleep duration measures indicated that our sample did not meet the average benchmark of at least 7 hours sleep and sleep quality was only in a medium range. This was also indicated by the PSQI, with a retrospectively reported sleep duration of *M* = 6.77 hours (*SD* = 0.84) for the last 4 weeks and a total PSQI score of *M* = 5.19 (*SD* = 2.19). Subjective and fitbit-measured sleep duration were highly correlated and negatively associated with participants’ age. Both sleep duration measures were positively associated with sleep quality in the MCII + SH group only. Participants’ average stress levels were negatively associated with the average in all sleep outcomes, though this association was only statistically significant in the MCII + SH group.

**Table 1.** Descriptive statistics separated by study group.

		1	2	3	4	5	6	*Mean* (*SD*)	ICC
1	Sex^a^		−0.13	−0.03	0.19	−0.04	−0.04	0.62 (0.49)	-
2	Age	0.08		−0.36*	−0.46*	0.10	−0.14	29.59 (4.45)	-
3	Subjective sleep duration (hours)	−0.10	−0.42*		0.72*	0.03	−0.19	6.75 (0.60)	0.201
4	Fitbit measured sleep duration (hours)	0.00	−0.38*	0.77*		−0.22	−0.17	6.73 (0.53)	0.131
5	Sleep quality^b^	0.17	−0.16	0.39*	0.44*		−0.11	54.92 (10.83)	0.297
6	Stress^b^	−0.04	0.23	−0.47*	−0.50*	−0.43*		42.09 (9.44)	0.136
	Mean (SD)	0.71 (0.46)	30.29 (3.72)	6.89 (0.56)	6.83 (0.59)	55.95 (13.78)	42.92 (13.28)		
	ICC	-	-	0.148	0.136	0.380	0.272		

Table depicts descriptive statistics on the between-person level.

Estimates in the upper diagonal depict estimates in the SH group (sleep hygiene), estimates in the lower diagonal the estimates in the MCII + SH group (mental contrasting with implementation intentions + sleep hygiene). Data for sleep outcomes and self-reported stress were aggregated across baseline and post-intervention weeks. ICC = intra-class correlation.

a 0 = male, 1 = female; ^b^0–100, higher scores indicating higher stress/sleep quality; *p < 0.05. *N* = 41 (MCII + SH); *N* = 39 (SH).

### Intervention effects

#### Preregistered analyses

Three multilevel models were estimated with fitbit-measured sleep duration, subjective sleep duration, and sleep quality as dependent variables, respectively. Results (Table 2, upper half) showed that, contrary to our hypotheses, there was no statistically meaningful group*week interaction for either sleep parameter, *p* > 0.767 for all. Hence, the MCII + SH group and the SH group did not differ in change in the sleep parameters from week 1 to week 2. Moreover, participants slept longer, *b* = 0.448 (fitbit-measured), *b* = 0.610 (subjective), *p* < 0.001, for both, and reported higher sleep quality, *b* = 6.967, *p* < 0.001, on the weekends compared to during the week. To examine overall changes we re-ran the models without the group*week interaction. Findings revealed no meaningful change in fitbit-measured sleep duration, *b* = 0.111, *p* = 0.109, but an increase in subjective sleep duration, *b* = 0.172, *p* = 0.007, and sleep quality, *b* = 2.977, *p* = 0.003, from baseline to post-intervention (aggregated across both groups). Sleep outcomes by study group during baseline and post-intervention period are depicted in Figure S1 (Supplement).

**Table 2.** Multilevel models: intervention effects and associations between daily stress and daily sleep parameters.

Intervention effects	Fitbit-measured sleep duration	Subjective sleep duration	Sleep quality
*Fixed effects*			
Intercept	6.484** (0.132)	6.527** (0.135)	51.279** (2.698)
Day of week^a^	0.448** (0.077)	0.610** (0.070)	6.969** (1.034)
Gender^b^	0.108 (0.133)	−0.080 (0.138)	1.942 (2.915)
Group^c^	0.089 (0.143)	0.166 (0.145)	0.804 (2.797)
Week^d^	0.111 (0.099)	0.191* (0.091)	2.930* (1.420)
Group * Week	−0.000 (0.139)	−0.037 (0.127)	0.090 (1.981)
*Random effects (standard deviations)*			
Intercept	0.464	0.507	10.970
Week	0.000	0.000	2.940
Residual (Level 1)	1.151	1.055	15.565
Associations stress and sleep	Fitbit-measured sleep duration	Subjective sleep duration	Sleep quality
*Fixed effects*			
Intercept	6.568** (0.102)	6.687**(0.108)	53.226** (2.288)
Day of week^a^	0.445** (0.079)	0.595** (0.072)	6.599** (1.065)
Gender^b^	0.129 (0.122)	−0.059 (0.130)	2.038 (2.785)
*Within-person effect*			
Stress	−0.000 (0.002)	−0.003 (0.002)	−0.060* (0.026)
*Between-person effect*			
Stress	−0.017** (0.005)	−0.017* (0.005)	−0.343* (0.115)
*Random effects (standard deviations)*			
Intercept	0.414	0.470	11.017
Stress	0.007	0.000	0.059
Residual (Level 1)	1.145	1.056	15.666

Table depicts point estimates of unstandardized coefficients (standard errors of fixed effects in parentheses). Number of observations = 1088–1112; number of participants = 80.

a 0 = weekday, 1 = weekend; ^b^0 = male, 1 = female; ^c^0 = SH group, 1 = MCII+SH group; ^d^0 = before intervention, 1 = after intervention.

* *p* < 0.05; **p < 0.001.

#### Exploratory analyses

To account for the possibility of gradual improvement of the sleep parameters after the intervention, we conducted piecewise growth models. Specifically, two variables were created to code the number of the study days. The first variable (time to intervention) counted the number of days to the first night after the intervention. The first day was coded as −7, with increments of one for each study day until day 8 (the night after the intervention); all days in the second week were coded as 0 for this variable. The second time-variable (time since intervention) was coded as zero for the first 8 days, with 1 on day 9, and counted up until +6 on day 14. These two time-variables and the interaction of these time-variables with group were entered in addition to the predictors in the model above. This model allows to estimate (a) gradual changes in sleep parameters before the intervention (and differences in this change between groups), (b) gradual changes in sleep parameters after the intervention (and differences in this change between groups), and (c) an abrupt change from day 7 to 8 (and differences in this change between groups). None of the change parameters or the interaction of change parameters with group were statistically significant, *p* > 0.107 for all (Supplement, Table S1). Hence, there was no evidence for gradual or abrupt changes (or group differences) of the three sleep parameters.

In an additional model, we examined change in the sleep parameters combined across the two groups. To that end, we re-estimated the pricewise growth models above, but removed the group variables (Supplement, Table S2). For subjective sleep duration and sleep quality, none of the gradual change parameters or the abrupt change parameter was significant, *p* > 0.154 for all. For fitbit-measured sleep duration, there was some evidence for an abrupt increase from the night before to after the intervention, *b* = 0.406, *p* = 0.012.

In further exploratory analyses, we examined if there was evidence for an intervention effect on subjective stress. Using subjective stress as the dependent variable in the same models (equations (1)–(4)) revealed no main effects of group or study week, and no significant group*week interaction, *p* > 0.390 for all. In a model without the group*week interaction, the main effect of week was not significant either, *b* = 0.020, *p* = 0.989.

Lastly, in further non-pre registered analyses, we examined if the two groups differed in the CAR from before to after the intervention. We examined intervention effects by predicting CAR from study week, group, and the group*week interaction in a multilevel model. As covariates on the between-person level, we included gender, intake of hormonal contraceptives, age, and body mass index. On the within-person level, we included consumption of drinks and the average activity level reported during the CAR assessment period. The main effects of group, *b* = 38.76, *p* = 0.322, and week, *b* = –36.77, *p* = 0.180, were not significant, and neither was the group*week interaction, *b* = 14.28, *p* = 0.715. Sensitivity analyses using only data of days when the first saliva sample was provided no later than 5 minutes after awakening did not change the pattern of results. Hence, there was no evidence for (group differences in) changes in the CAR.

### Association of stress with sleep parameters

We examined if daily stress was associated with the three sleep parameters on the within-person level (e.g. “Is sleep quality lower on nights following days with higher than usual stress?”) and the between person level (e.g. “Do participants with higher average stress levels report worse sleep quality on average?”). To that end, three multilevel models were estimated. Results consistently showed significant associations of daily stress with all three sleep parameters on the between-person level (Table 2, lower half): Participants who reported higher average stress slept shorter, both assessed via Fitbit, *b* = –0.017, *p* < 0.001, and via subjective sleep duration, *b* = –0.017, *p* = 0.003, and they also reported lower sleep quality on average, *b* = –0.343, *p* = 0.004. On the within-person level, only the association of stress with next night sleep quality was statistically meaningful, *b* = –0.060, *p* = 0.023, meaning that days with higher than usual stress tend to be followed by nights with lower sleep quality. There was no within-person association of stress with next night’s fitbit-measured sleep duration, *b* = 0.000, *p* = 0.903, or subjective sleep duration, *b* = –0.003, *p* = –0.059. Although there was no intervention effect on daily subjective stress or the CAR, we found a decrease in self-reported stress from prior to baseline to a 3-week follow-up (Supplement, Figure S2).

## Discussion

Our randomized-controlled study did not support the effectiveness of an MCII intervention component over sleep hygiene information in a sample of early career researchers. Across all participants, results indicated an increase in sleep quality and subjective (but not objective) sleep duration from baseline to post-intervention period. Contrary to our preregistered hypotheses, there was no statistically meaningful group*week interaction for either sleep parameter indicating that the intervention group (MCII + SH) and the active control group (SH) did not differ in change in the sleep parameters from baseline to post-intervention week.

Regarding daily stress, our hypothesis was partially confirmed, as significant associations of stress with all three sleep parameters on the between-person level were found: Participants with higher average stress slept shorter (objectively and subjectively) and reported lower sleep quality. On the within-person level, days with higher than usual stress were followed by nights with lower sleep quality, confirming the results of Åkerstedt et al. (2012), who found that stress at bedtime predicted subjective sleep quality. However, our data did not reveal associations of stress with next night’s sleep duration measures. High quality micro-longitudinal studies on sleep duration and stress or negative affect are still rare, with recent studies showing mixed evidence (Sin et al., 2017; Slavish et al., 2021). Moreover, despite a clear temporal ordering in our study (stress was assessed the evening before the assessment of subjective sleep quality), the stress-sleep association remains to be subject to potential third-variable confounding (Neubauer and Schmiedek, 2020).

In line with the results on sleep outcomes, exploratory analyses regarding effects of the MCII intervention on the CAR did not show any significant effects. Thus, changes in the CAR from pre- to post-intervention were not different between study groups. Furthermore, overall changes in the CAR (across all participants) were not significant either.

A possible explanation for the null-findings regarding the additional effectiveness of MCII on sleep outcomes is the short time-frame of the present study. For instance, Stadler et al. (2010) reported no advantage of MCII compared to an information only-condition on eating behavior during the first 2 months. However, they similarly observed improvements across the total sample. After 4 months, the superiority of MCII emerged and even increased at 2-year follow-up, whereas the information only-condition returned to baseline level. Potentially, interventions implemented at a single time point might be less effective and their effects dissipate rather quickly. In a different context, Breitwieser et al. (2022) found that the effectiveness of implementation intentions to foster self-regulated learning in medical students built up over time. Hence, booster sessions could be required for MCII to unfold full potential.

Moreover, the suitability of MCII addressing sleep behavior needs to be discussed, as this study was the first to expand MCII on sleep research at the point of data collection. The approach to use MCII to improve sleep outcomes is based on the assumption that insufficient sleep results (at least partially) from self-regulatory failure. This assumption seems justified if sleep problems are due to factors implying a lack of self-control, such as “bedtime procrastination,” defined as going to bed later than intended without any legitimate reason and although negative consequences are expected (Kroese et al., 2016). The recent study on the effects of MCII on bedtime procrastination by Valshtein et al. (2020) found positive effects in terms of a reduced discrepancy between intended and actual bedtime. However, and in line with our results, self-reported sleep duration was not affected by MCII, as participants did not actually go to bed earlier or wake up later, but only revised their planned bedtimes. With respect to bedtime procrastination, our participants reported moderate levels on average (*M* = 3.13, *SD* = 0.67; 5-point scales from 1 = never to 5 = always) which is slightly higher than in a representative Dutch sample of Kroese et al. (2016); *M* = 2.8, *SD* = 0.8), indicating that the phenomenon is indeed commonly experienced.

Aside from procrastination, it could be assumed that if sleep issues are due to other factors such as rumination or pre-sleep arousal, MCII might not be as effective. Health behaviors such as exercise or nutrition can be influenced more directly, whereas sleep outcomes are more difficult to change as they vary/differ in their degree of controllability. Trying harder is not necessarily the most successful approach when trying to fall asleep, and sleep problems cannot always be solved by exerting higher self-control. Another explanation for the null-finding might be a mediating role of energization regarding the MCII effect on goal commitment proposed by Oettingen et al. (2009), who assessed energization both via systolic blood pressure and self-reports. Whereas higher energization might be helpful to achieve most health-related goals, this might not be true for sleep outcomes, as falling asleep is associated with a decrease in systolic blood pressure (Calhoun and Harding, 2010). In order to evaluate generalizability, it should be noted that by using the recommended cut-off score of 5 for the PSQI, 41% of our sample can be described as “poor sleepers,” although nobody reported having received a diagnosis of insomnia or other sleep-related disorders. This is slightly higher than in a representative German sample (36%; Hinz et al., 2017), but not unexpected given the focus on sleep in the study description.

In addition to the short time frame, several limitations have to be considered. First, our design lacked a passive control group without intervention, making it impossible to attribute the positive overall increases to either sleep hygiene information (+MCII) or to the intensive self-monitoring with diaries and the Fitbit. Second, the study aim was rather obvious, which could have resulted in demand effects, which is supported by the fact that only self-reported measures, but not fitbit-measured sleep duration increased over the study period. However, also for fitbit-measured sleep duration, there was evidence for an abrupt increase from the night before to after the intervention. Third, our findings are based on a sample of university employees with relatively high degrees of flexibility in work schedules and findings may only be valid for populations with comparable job-related characteristics. Fourth, regarding CAR, we did not use objective methods to verify awakening or sampling times. Participants did not receive prompts to remind them of S2 or S3, but were instructed to set timers. Although participants received detailed information on the importance of adherence and although we applied strict accuracy margins in sensitivity analyses, this could have led to inaccurate CAR estimates. Furthermore, we were only able to assess the CAR on 2 consecutive days for each study period. Given the high variability of the CAR we therefore assessed important trait and state covariates to exclude CARs or control for them.

Strengths of the present study include a strong active control condition, allowing detection of possible effects of MCII over a commonly used alternative (SH), a standardized intervention protocol allowing replication, a low dropout-rate, and the combined and intensive ambulatory assessment of daily self-report, CAR-assessment and fitbit-measured data. According to a meta-analysis (Haghayegh et al., 2019) newer models like the Fitbit Alta outperform early-generation models in terms of sensitivity and specificity. Although not recommended for accurate point estimates, validation studies indicate that they are able to correctly identify intervention effects (Dickinson et al., 2016). In general, assessments of objective sleep parameters serve as a valuable complement, as relevant parameters such as time of falling asleep or nighttime awakenings may not be accurate in self-reports due to low levels of consciousness.

To conclude, our study did not find incremental effects of a MCII component over sleep hygiene (and self-monitoring) in a short time period as sleep quality and (subjective) sleep duration increased in both groups. The CAR did neither reveal changes nor differences between groups. Regarding subjective stress, associations with daily sleep parameters were largely confirmed. Future research should include booster sessions and evaluate MCII effects in the longer run. Additionally, the content of the implementations could be matched with a more diverse range of sleep-related outcomes. In our case, we learned from a workshop with 22 of our participants after data collection, that some participants perceived MCII as helpful to achieve their individual sleep-related goals (e.g. sleep onset latency, sleep schedule, sleep hygiene practice), but that this intervention effect was not reflected in our pre-defined outcomes sleep duration and quality.

Aside from the need to disentangle effects of single behavior change components, a better understanding of the causes regarding insufficient sleep among specific target groups and their degree of controllability is required to develop individually targeted interventions. Additionally, moderators and mediators that have been recently linked to self-regulation, stress and sleep should be addressed, that is, detachment from work, rumination, or media/smartphone use (e.g. Zhang and Wu, 2020). Lastly, our study contributes to behavior change literature in the field of sleep research through the development and testing of a theory-guided MCII intervention.

## Research Data

sj-csv-12-hpq-10.1177_13591053231159168 – Supplemental material for Effects of mental contrasting on sleep and associations with stress: A randomized controlled trial Click here for additional data file. sj-csv-12-hpq-10.1177_13591053231159168 for Effects of mental contrasting on sleep and associations with stress: A randomized controlled trial by Laura I Schmidt, Andreas B Neubauer, Martin Stoffel, Beate Ditzen, Jana Schirmaier, Claudia Farrenkopf and Monika Sieverding in Journal of Health Psychology This article is distributed under the terms of the Creative Commons Attribution 4.0 License ( http://www.creativecommons.org/licenses/by/4.0/) which permits any use, reproduction and distribution of the work without further permission provided the original work is attributed as specified on the SAGE and Open Access pages ( https://us.sagepub.com/en-us/nam/open-access-at-sage).

sj-csv-13-hpq-10.1177_13591053231159168 – Supplemental material for Effects of mental contrasting on sleep and associations with stress: A randomized controlled trial Click here for additional data file. sj-csv-13-hpq-10.1177_13591053231159168 for Effects of mental contrasting on sleep and associations with stress: A randomized controlled trial by Laura I Schmidt, Andreas B Neubauer, Martin Stoffel, Beate Ditzen, Jana Schirmaier, Claudia Farrenkopf and Monika Sieverding in Journal of Health Psychology This article is distributed under the terms of the Creative Commons Attribution 4.0 License ( http://www.creativecommons.org/licenses/by/4.0/) which permits any use, reproduction and distribution of the work without further permission provided the original work is attributed as specified on the SAGE and Open Access pages ( https://us.sagepub.com/en-us/nam/open-access-at-sage).

sj-csv-14-hpq-10.1177_13591053231159168 – Supplemental material for Effects of mental contrasting on sleep and associations with stress: A randomized controlled trial Click here for additional data file. sj-csv-14-hpq-10.1177_13591053231159168 for Effects of mental contrasting on sleep and associations with stress: A randomized controlled trial by Laura I Schmidt, Andreas B Neubauer, Martin Stoffel, Beate Ditzen, Jana Schirmaier, Claudia Farrenkopf and Monika Sieverding in Journal of Health Psychology This article is distributed under the terms of the Creative Commons Attribution 4.0 License ( http://www.creativecommons.org/licenses/by/4.0/) which permits any use, reproduction and distribution of the work without further permission provided the original work is attributed as specified on the SAGE and Open Access pages ( https://us.sagepub.com/en-us/nam/open-access-at-sage).

sj-DOCX-16-hpq-10.1177_13591053231159168 – Supplemental material for Effects of mental contrasting on sleep and associations with stress: A randomized controlled trial Click here for additional data file. Supplemental material, sj-DOCX-16-hpq-10.1177_13591053231159168 for Effects of mental contrasting on sleep and associations with stress: A randomized controlled trial by Laura I Schmidt, Andreas B Neubauer, Martin Stoffel, Beate Ditzen, Jana Schirmaier, Claudia Farrenkopf and Monika Sieverding in Journal of Health Psychology

sj-inp-10-hpq-10.1177_13591053231159168 – Supplemental material for Effects of mental contrasting on sleep and associations with stress: A randomized controlled trial Click here for additional data file. sj-inp-10-hpq-10.1177_13591053231159168 for Effects of mental contrasting on sleep and associations with stress: A randomized controlled trial by Laura I Schmidt, Andreas B Neubauer, Martin Stoffel, Beate Ditzen, Jana Schirmaier, Claudia Farrenkopf and Monika Sieverding in Journal of Health Psychology This article is distributed under the terms of the Creative Commons Attribution 4.0 License ( http://www.creativecommons.org/licenses/by/4.0/) which permits any use, reproduction and distribution of the work without further permission provided the original work is attributed as specified on the SAGE and Open Access pages ( https://us.sagepub.com/en-us/nam/open-access-at-sage).

sj-inp-11-hpq-10.1177_13591053231159168 – Supplemental material for Effects of mental contrasting on sleep and associations with stress: A randomized controlled trial Click here for additional data file. sj-inp-11-hpq-10.1177_13591053231159168 for Effects of mental contrasting on sleep and associations with stress: A randomized controlled trial by Laura I Schmidt, Andreas B Neubauer, Martin Stoffel, Beate Ditzen, Jana Schirmaier, Claudia Farrenkopf and Monika Sieverding in Journal of Health Psychology This article is distributed under the terms of the Creative Commons Attribution 4.0 License ( http://www.creativecommons.org/licenses/by/4.0/) which permits any use, reproduction and distribution of the work without further permission provided the original work is attributed as specified on the SAGE and Open Access pages ( https://us.sagepub.com/en-us/nam/open-access-at-sage).

sj-inp-9-hpq-10.1177_13591053231159168 – Supplemental material for Effects of mental contrasting on sleep and associations with stress: A randomized controlled trial Click here for additional data file. sj-inp-9-hpq-10.1177_13591053231159168 for Effects of mental contrasting on sleep and associations with stress: A randomized controlled trial by Laura I Schmidt, Andreas B Neubauer, Martin Stoffel, Beate Ditzen, Jana Schirmaier, Claudia Farrenkopf and Monika Sieverding in Journal of Health Psychology This article is distributed under the terms of the Creative Commons Attribution 4.0 License ( http://www.creativecommons.org/licenses/by/4.0/) which permits any use, reproduction and distribution of the work without further permission provided the original work is attributed as specified on the SAGE and Open Access pages ( https://us.sagepub.com/en-us/nam/open-access-at-sage).

sj-pdf-15-hpq-10.1177_13591053231159168 – Supplemental material for Effects of mental contrasting on sleep and associations with stress: A randomized controlled trial Click here for additional data file. sj-pdf-15-hpq-10.1177_13591053231159168 for Effects of mental contrasting on sleep and associations with stress: A randomized controlled trial by Laura I Schmidt, Andreas B Neubauer, Martin Stoffel, Beate Ditzen, Jana Schirmaier, Claudia Farrenkopf and Monika Sieverding in Journal of Health Psychology This article is distributed under the terms of the Creative Commons Attribution 4.0 License ( http://www.creativecommons.org/licenses/by/4.0/) which permits any use, reproduction and distribution of the work without further permission provided the original work is attributed as specified on the SAGE and Open Access pages ( https://us.sagepub.com/en-us/nam/open-access-at-sage).

sj-R-1-hpq-10.1177_13591053231159168 – Supplemental material for Effects of mental contrasting on sleep and associations with stress: A randomized controlled trial Click here for additional data file. sj-R-1-hpq-10.1177_13591053231159168 for Effects of mental contrasting on sleep and associations with stress: A randomized controlled trial by Laura I Schmidt, Andreas B Neubauer, Martin Stoffel, Beate Ditzen, Jana Schirmaier, Claudia Farrenkopf and Monika Sieverding in Journal of Health Psychology This article is distributed under the terms of the Creative Commons Attribution 4.0 License ( http://www.creativecommons.org/licenses/by/4.0/) which permits any use, reproduction and distribution of the work without further permission provided the original work is attributed as specified on the SAGE and Open Access pages ( https://us.sagepub.com/en-us/nam/open-access-at-sage).

sj-R-2-hpq-10.1177_13591053231159168 – Supplemental material for Effects of mental contrasting on sleep and associations with stress: A randomized controlled trial Click here for additional data file. sj-R-2-hpq-10.1177_13591053231159168 for Effects of mental contrasting on sleep and associations with stress: A randomized controlled trial by Laura I Schmidt, Andreas B Neubauer, Martin Stoffel, Beate Ditzen, Jana Schirmaier, Claudia Farrenkopf and Monika Sieverding in Journal of Health Psychology This article is distributed under the terms of the Creative Commons Attribution 4.0 License ( http://www.creativecommons.org/licenses/by/4.0/) which permits any use, reproduction and distribution of the work without further permission provided the original work is attributed as specified on the SAGE and Open Access pages ( https://us.sagepub.com/en-us/nam/open-access-at-sage).

sj-R-3-hpq-10.1177_13591053231159168 – Supplemental material for Effects of mental contrasting on sleep and associations with stress: A randomized controlled trial Click here for additional data file. sj-R-3-hpq-10.1177_13591053231159168 for Effects of mental contrasting on sleep and associations with stress: A randomized controlled trial by Laura I Schmidt, Andreas B Neubauer, Martin Stoffel, Beate Ditzen, Jana Schirmaier, Claudia Farrenkopf and Monika Sieverding in Journal of Health Psychology This article is distributed under the terms of the Creative Commons Attribution 4.0 License ( http://www.creativecommons.org/licenses/by/4.0/) which permits any use, reproduction and distribution of the work without further permission provided the original work is attributed as specified on the SAGE and Open Access pages ( https://us.sagepub.com/en-us/nam/open-access-at-sage).

sj-R-4-hpq-10.1177_13591053231159168 – Supplemental material for Effects of mental contrasting on sleep and associations with stress: A randomized controlled trial Click here for additional data file. sj-R-4-hpq-10.1177_13591053231159168 for Effects of mental contrasting on sleep and associations with stress: A randomized controlled trial by Laura I Schmidt, Andreas B Neubauer, Martin Stoffel, Beate Ditzen, Jana Schirmaier, Claudia Farrenkopf and Monika Sieverding in Journal of Health Psychology This article is distributed under the terms of the Creative Commons Attribution 4.0 License ( http://www.creativecommons.org/licenses/by/4.0/) which permits any use, reproduction and distribution of the work without further permission provided the original work is attributed as specified on the SAGE and Open Access pages ( https://us.sagepub.com/en-us/nam/open-access-at-sage).

sj-txt-5-hpq-10.1177_13591053231159168 – Supplemental material for Effects of mental contrasting on sleep and associations with stress: A randomized controlled trial Click here for additional data file. sj-txt-5-hpq-10.1177_13591053231159168 for Effects of mental contrasting on sleep and associations with stress: A randomized controlled trial by Laura I Schmidt, Andreas B Neubauer, Martin Stoffel, Beate Ditzen, Jana Schirmaier, Claudia Farrenkopf and Monika Sieverding in Journal of Health Psychology This article is distributed under the terms of the Creative Commons Attribution 4.0 License ( http://www.creativecommons.org/licenses/by/4.0/) which permits any use, reproduction and distribution of the work without further permission provided the original work is attributed as specified on the SAGE and Open Access pages ( https://us.sagepub.com/en-us/nam/open-access-at-sage).

sj-txt-6-hpq-10.1177_13591053231159168 – Supplemental material for Effects of mental contrasting on sleep and associations with stress: A randomized controlled trial Click here for additional data file. sj-txt-6-hpq-10.1177_13591053231159168 for Effects of mental contrasting on sleep and associations with stress: A randomized controlled trial by Laura I Schmidt, Andreas B Neubauer, Martin Stoffel, Beate Ditzen, Jana Schirmaier, Claudia Farrenkopf and Monika Sieverding in Journal of Health Psychology This article is distributed under the terms of the Creative Commons Attribution 4.0 License ( http://www.creativecommons.org/licenses/by/4.0/) which permits any use, reproduction and distribution of the work without further permission provided the original work is attributed as specified on the SAGE and Open Access pages ( https://us.sagepub.com/en-us/nam/open-access-at-sage).

sj-txt-7-hpq-10.1177_13591053231159168 – Supplemental material for Effects of mental contrasting on sleep and associations with stress: A randomized controlled trial Click here for additional data file. sj-txt-7-hpq-10.1177_13591053231159168 for Effects of mental contrasting on sleep and associations with stress: A randomized controlled trial by Laura I Schmidt, Andreas B Neubauer, Martin Stoffel, Beate Ditzen, Jana Schirmaier, Claudia Farrenkopf and Monika Sieverding in Journal of Health Psychology This article is distributed under the terms of the Creative Commons Attribution 4.0 License ( http://www.creativecommons.org/licenses/by/4.0/) which permits any use, reproduction and distribution of the work without further permission provided the original work is attributed as specified on the SAGE and Open Access pages ( https://us.sagepub.com/en-us/nam/open-access-at-sage).

sj-txt-8-hpq-10.1177_13591053231159168 – Supplemental material for Effects of mental contrasting on sleep and associations with stress: A randomized controlled trial Click here for additional data file. sj-txt-8-hpq-10.1177_13591053231159168 for Effects of mental contrasting on sleep and associations with stress: A randomized controlled trial by Laura I Schmidt, Andreas B Neubauer, Martin Stoffel, Beate Ditzen, Jana Schirmaier, Claudia Farrenkopf and Monika Sieverding in Journal of Health Psychology This article is distributed under the terms of the Creative Commons Attribution 4.0 License ( http://www.creativecommons.org/licenses/by/4.0/) which permits any use, reproduction and distribution of the work without further permission provided the original work is attributed as specified on the SAGE and Open Access pages ( https://us.sagepub.com/en-us/nam/open-access-at-sage).

## Notes

1. An additional hypothesis was preregistered regarding a self-developed measurement of sleep hygiene behavior. It could not be tested due to a programing error in the online questionnaire resulting in erroneous time frames for the respective items. Study preregistration information: https://osf.io/q2te4

2. Time stamps of the online questionnaires indicated that on 48 nights the time between completing the evening questionnaire and the next morning questionnaire was less than 1 hour. Re-running the main analyses after removing these 48 observations did not change the pattern of results, however.
