# Supplementary material for: Effects of mental contrasting on sleep and associations with stress: A randomized controlled trial
Source: J Health Psychol. 2023 Mar 15;28(11):1057–71. doi: 10.1177/13591053231159168 (PMC10492430; doi:10.1177/13591053231159168)
Supplement: sj-DOCX-16-hpq-10.1177_13591053231159168 – Supplemental material for Effects of mental contrasting on sleep and associations with stress: A randomized controlled trial [file sj-DOCX-16-hpq-10.1177_13591053231159168.DOCX]

**Supplement**

Table S1

Results of exploratory piecewise growth models including interaction terms.

|  | Fitbit measured sleep duration | Subjective Sleep duration | Sleep Quality |
| --- | --- | --- | --- |
| **Fixed Effects** |  |  |  |
| Intercept | 6.468** (0.212) | 6.620** (0.202) | 50.921** (3.646) |
| Day of week^a^ | 0.542** (0.087) | 0.575** (0.080) | 6.198** (1.173) |
| Gender^b^ | 0.113 (0.128) | -0.074 (0.137) | 1.042 (2.863) |
| Group^c^ | -0.213 (0.266) | 0.162 (0.245) | 2.889 (4.311) |
| Time to intervention | 0.003 (0.037) | 0.022 (0.033) | -0.033 (0.511) |
| Tine since intervention | -0.061 (0.038) | -0.010 (0.034) | 0.485 (0.494) |
| Week^d^ | 0.275 (0.221) | 0.135 (0.195) | 1.623 (2.971) |
| Group x Time to intervention | -0.076 (0.050) | -0.001 (0.045) | 0.501 (0.692) |
| Group x Time since intervention | 0.016 (0.051) | 0.042 (0.045) | 0.039 (0.662) |
| Group x Week | 0.259 (0.296) | -0.160 (0.259) | -2.039 (3.957) |
| **Random Effects**  (Standard Deviations) |  |  |  |
| Intercept | 0.683 | 0.638 | 14.094 |
| Time to intervention | 0.051 | 0.035 | 0.995 |
| Time since intervention | 0.056 | 0.000 | 0.000 |
| Week | 0.436 | 0.154 | 5.426 |
| Residual (Level 1) | 1.143 | 1.054 | 15.490 |

*Note.* Table depicts point estimates of unstandardized coefficients (standard errors of fixed effects in parentheses). Number of observations = 1,101 – 1,112; number of participants = 80. Time to intervention was coded as -7 on the first study day and increased by one for each study day until day 8 (all days on or after day eight were coded as 0). Time since intervention was coded as 0 until day 8, and increased by one on each day (day 9 = 1, day 10 = 2 ... day 14 = 6).

^a^0 = weekday, 1 = weekend; ^b^0 = male, 1 = female; ^c^0 = SH group, 1 = MCII+SH group; ^d^0 = before intervention, 1 = after intervention.

**p* < .05; ***p* < .001

**Supplement**

Table S2

Results of exploratory piecewise growth models without interaction terms.

|  | Fitbit measured sleep duration | Subjective Sleep duration | Sleep Quality |
| --- | --- | --- | --- |
| **Fixed Effects** |  |  |  |
| Intercept | 6.353** (0.168) | 6.693** (0.162) | 52.318** (2.970) |
| Day of week^a^ | 0.542** (0.087) | 0.575** (0.080) | 6.196** (1.172) |
| Gender^b^ | 0.125 (0.128) | -0.059 (0.136) | 1.170 (2.846) |
| Time to intervention | -0.035 (0.027) | 0.021 (0.024) | 0.224 (0.370) |
| Tine since intervention | -0.052 (0.027) | 0.011 (0.024) | 0.506 (0.354) |
| Week^d^ | 0.406* (0.161) | 0.053 (0.143) | 0.580 (2.162) |
| **Random Effects**  (Standard Deviations) |  |  |  |
| Intercept | 0.671 | 0.639 | 13.934 |
| Time to intervention | 0.048 | 0.033 | 1.055 |
| Time since intervention | 0.055 | 0.000 | 0.000 |
| Week | 0.415 | 0.154 | 5.327 |
| Residual (Level 1) | 1.144 | 1.053 | 15.473 |

*Note.* Table depicts point estimates of unstandardized coefficients (standard errors of fixed effects in parentheses). Number of observations = 1,101 – 1,112; number of participants = 80. Time to intervention was coded as -7 on the first study day and increased by one for each study day until day 8 (all days on or after day eight were coded as 0). Time since intervention was coded as 0 until day 8, and increased by one on each day (day 9 = 1, day 10 = 2 ... day 14 = 6).

^a^0 = weekday, 1 = weekend; ^b^0 = male, 1 = female; ^c^0 = SH group, 1 = MCII+SH group; ^d^0 = before intervention, 1 = after intervention.

**p* < .05; ***p* < .001

**Supplement**


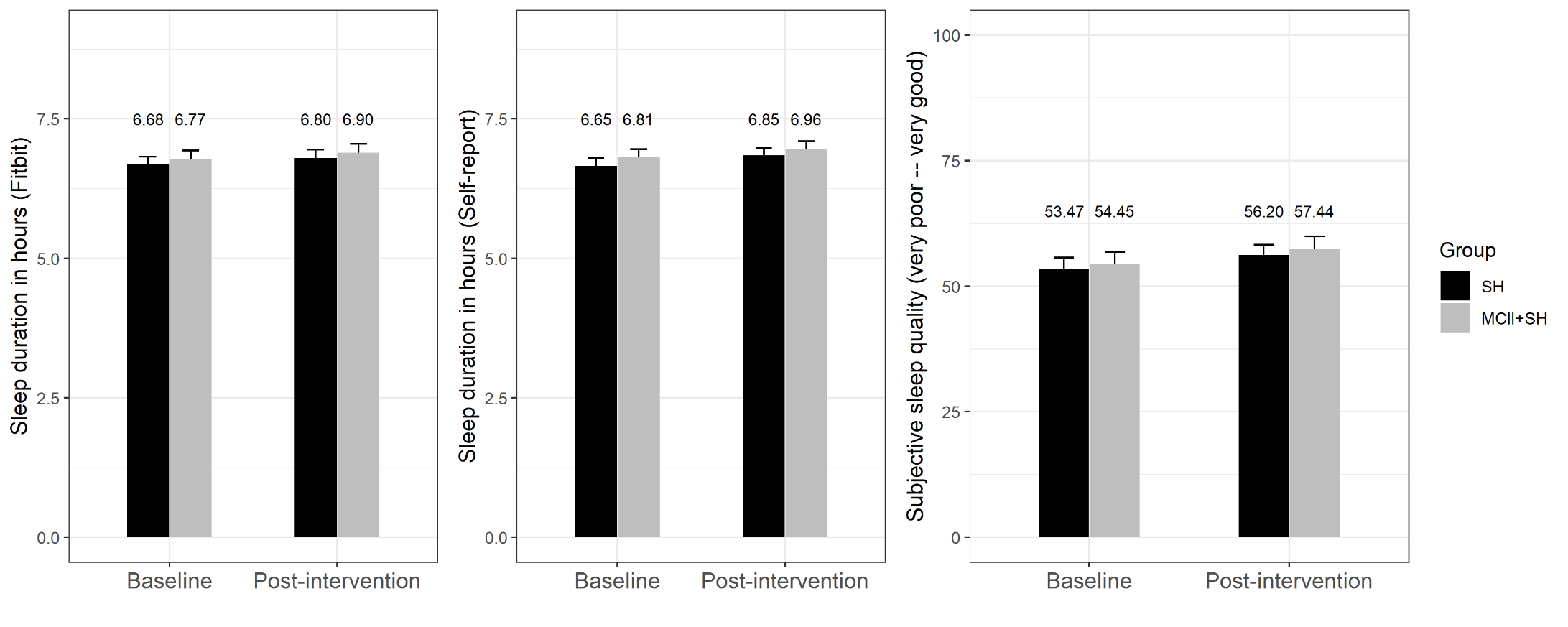


Figure S1: Sleep outcomes by study group during baseline and post-intervention period.

Note: mean sleep outcomes aggregated over 7 nights (baseline) and 7 nights (post-intervention). Error bars denote 95% bootstrap confidence intervals. MCII+SH = mental contrasting with implementation intentions + sleep hygiene (grey bars), SH = sleep hygiene only (black bars)

**Supplement**


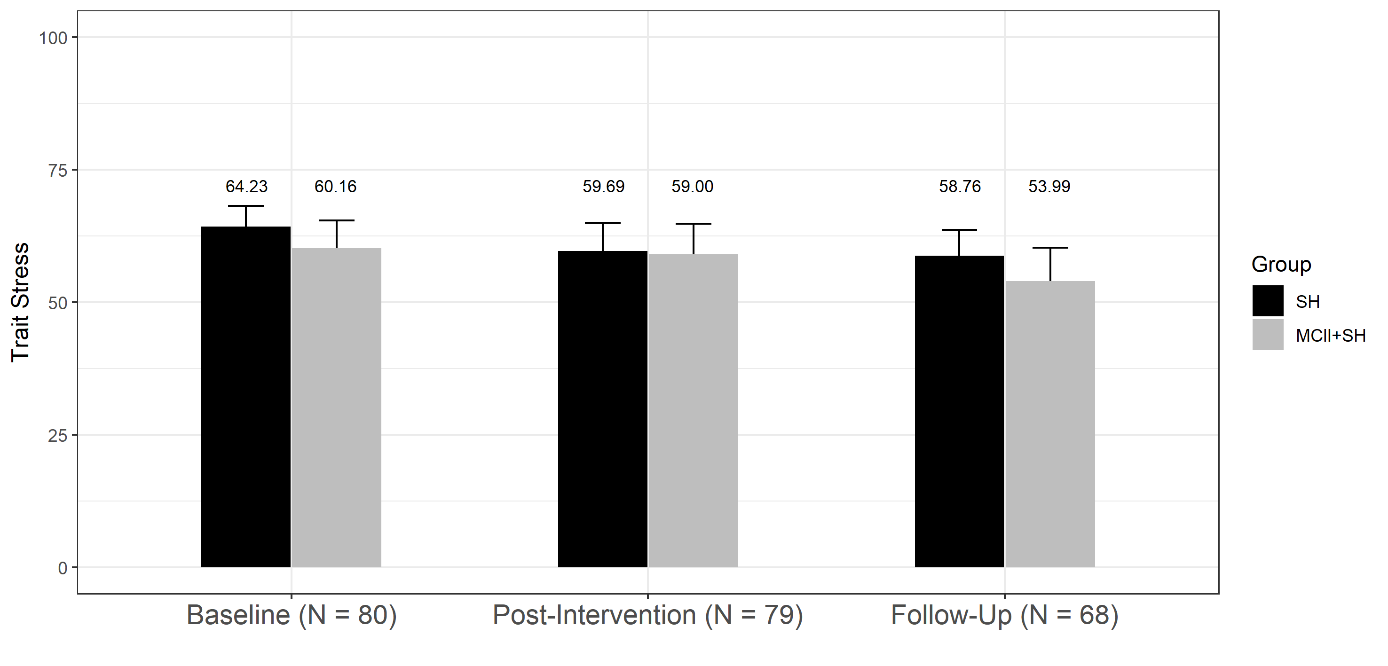


Figure S2: Self-reported stress by study group at baseline, post-intervention period and follow-up.

*Note.* MCII+SH = mental contrasting with implementation intentions + sleep hygiene (grey bars), SH = sleep hygiene only (black bars). Error bars indicate 95% bootstrap confidence intervals.

Stress was measured before the beginning of the baseline-week (Baseline), after the post-intervention-week (Post-Intervention) and at a Follow Up three weeks after the post-intervention week (Follow-Up). We assessed the Heidelberger Stress Index (HEI-STRESS) via online-questionnaire, with higher scores indicating higher stress, i.e. “over the last two weeks, how stressed did you feel due to your work from 0 (*not at all*) to 100 (*very stressed*).

Data were analyzed using a multilevel model with the repeated measures factor time (3 levels) and the between-person factor group (2 levels). The main effect of the factor time was statistically significant, *F*(2, 143) = 3.86, *p* = .023, the main effect of group, *F*(1, 78) = 1.00, *p* = .321, and the group by time interaction were not statistically significant, *F*(2, 143) = 0.49, *p* = .614. We followed up these results by inspecting post-hoc contrasts in a model excluding the interaction term. After adjusting for multiple comparisons using the Tukey method, only the difference between baseline and follow-up was statistically significant, *b* = 6.26, *p* = .017, indicating that across the two groups, stress levels were lower at follow-up than at baseline.
