# Supplementary material for: Effects of mental contrasting on sleep and associations with stress: A randomized controlled trial
Source: J Health Psychol. 2023 Mar 15;28(11):1057–71. doi: 10.1177/13591053231159168 (PMC10492430; doi:10.1177/13591053231159168)
Supplement: sj-pdf-15-hpq-10.1177_13591053231159168 – Supplemental material for Effects of mental contrasting on sleep and associations with stress: A randomized controlled trial [file sj-pdf-15-hpq-10.1177_13591053231159168.pdf]

# Readme PDF

Study preregistration information: https://osf.io/q2te4

Links to data, materials, and code:
https://osf.io/hq7mf/?view_only=e38bfaf5319d4e679487b1a40141e6b7

## Data and Syntax

### Data files:

"Cortisol sleepwell.csv": data set including cortisol variables (AUCi, AUCg) for the analyses of the saliva samples. Needed the analysis of the cortisol data (script 03_CAR analyses).

"person data.csv": data set including all baseline data, i.e. sociodemographic information health related information. Needed for all four analysis scripts (01, 02, 02s, 03).

"daily.csv": main data set including all data assessed in the daily diaries, i.e. sleep variables, stress, or daily control variables relevant for the cortisol analyses (i.e. caffeine). Needed for all four analysis scripts (01, 02, 02s, 03).

"data.dat": contains person data and daily data in Mplus readable format. Data file is created in the R script "02_main analyses sleep and stress".

### Syntax files:

R-File "01_descriptive statistics.R": running this code will produce all reported results in the manuscript in Table 1.

- ➔ See "01_descriptive statistics_results.txt" for the resulting R output

Mplus files "corr SD.inp", "corr SQ items.inp", and "corr stress items.inp": running this code will produce all reported results in the manuscript, section "Method, Measurements of the main study variables"

- ➔ See "corr SD.out", "corr SQ items.out", and "corr stress items.out" for the resulting Mplus outputs

R-File "02_main analyses sleep and stress.R": running this code will produce all reported results in the manuscript in the Results section, including Table 2, Table 3, Figure 2, Table S1, Table S2, and Figure S1. Not included in this script are analyses on the cortisol awakening response (CAR; see next script)

- ➔ See "02_main analyses sleep and stress_results.txt" for the resulting R output

R-File “03_CAR analyses.R” running this code will produce all results on the CAR analyses, which are reported in Results section of the manuscript

➔ See “03_CAR analyses_results.txt” for the resulting R output

Data were analyzed using R version 4.2.1 for Windows and Mplus Version 8.6 for Windows. Multilevel models were estimated using the nlme package, Version 3.1-157 (Pinheiro J et al., 2021). The following packages were used for data preparation, plotting, and basic analyses:

- emmeans (version 1.8.1-1)
- psych (version 2.2.)
- ggplot2 (version 3.3.6)
- grid (version 4.2.1)
- gridExtra (version 2.3)
- plyr (version 1.8.7)
- MplusAutomation (version 1.1.0)

**Log files:**

01_descriptive statistics_results.txt: R output resulting for running the R syntax “01_descriptive statistics_results.R”

02_main analyses sleep and stress_results.txt: R output resulting for running the R syntax “02_main analyses sleep and stress_results.R”

03_CAR analyses_results.txt: R output resulting for running the R syntax “03_CAR analyses_results.R”

02s_sensitivity analyses for prereg analyses_results.txt: R output resulting for running the R syntax “02s_sensitivity analyses for prereg analyses_results.R”

**Additional analysis:**

R-File 02s_sensitivity analyses for prereg analyses: running this code will produce results of sensitivity analyses for which individual data points were excluded, if timestamps indicated that the time between completing the evening questionnaire and the next morning questionnaire was less than one hour (see Footnote 2).

➔ See “02s_sensitivity analyses for prereg analyses_results.txt” for the resulting R output
